# Ear acupuncture with laser and needles in the treatment of anxiety in university students: a randomized clinical trial*

**DOI:** 10.1590/1980-220X-REEUSP-2024-0239en

**Published:** 2025-01-27

**Authors:** Ana Paula da Silva Lemos, Bárbara Guimarães Lourenço, Érika de Cássia Lopes Chaves, Luana Vieira Toledo, Tânia Couto Machado Chianca, Caroline de Castro Moura

**Affiliations:** 1 Universidade Federal de Viçosa, Programa de Pós-graduação em Ciências da Saúde, Viçosa, MG, Brazil.; 2 Universidade Federal de Viçosa, Departamento de Medicina e Enfermagem, Viçosa, MG, Brazil.; 3 Universidade Federal de Alfenas, Escola de Enfermagem, Alfenas, MG, Brazil.; 4 Universidade Federal de Minas Gerais, Departamento de Enfermagem Básica, Belo Horizonte, MG, Brazil.

**Keywords:** Acupuncture, Ear, Low-Level Light Therapy, Universities, Ansiedade, COVID-19, Acupuntura Auricular, Terapia por Luz de Baja Intensidad, Universidades, COVID-19.

## Abstract

**Objective:**

To compare the effectiveness of ear acupuncture with laser and needles in the treatment of anxiety in university students in the post-pandemic context of Covid-19, as well as to evaluate the possible symptoms or adverse reactions triggered by the interventions.

**Method:**

Randomized clinical trial carried out with 126 university students, allocated to the “Needle” (control) and “Laser” (experimental) groups. Five ear acupuncture sessions were performed. Assessments were performed before, after the end of treatment and seven days later (*follow up*), through the State Anxiety Inventory and the measurement of heart and respiratory rates. The *Generalized Estimating Equations* were used.

**Results:**

There was a reduction in anxiety levels in both groups, between the initial and final assessments, and initial and follow up. There was no difference between the groups in the final and follow up. Heart rate decreased in the Needle group between the final and follow up, and initial and follow up assessments; the Laser group remained constant over time and the groups did not differ from each other between the end and follow up. There was no change over time in respiratory rate in either group and they did not differ from each other in the final and follow up assessments. There was a higher occurrence of adverse reactions in the group that received the intervention with needles, despite the mild intensity.

**Conclusion:**

Both interventions were effective in reducing anxiety levels in university students. Brazilian Clinical Trials Registry RBR-8cxnvr2.

## RESUMEN

**Objetivo:**

Comparar la efectividad de la auriculoacupuntura con láser y agujas en el tratamiento de la ansiedad en estudiantes universitarios en el contexto pospandemia de Covid-19, así como evaluar posibles síntomas o reacciones adversas desencadenadas por las intervenciones.

**Método:**

Ensayo clínico aleatorizado realizado con 126 estudiantes universitarios, asignados a los grupos “Aguja” (control) y “Láser” (experimental). Se realizaron cinco sesiones de auriculoacupuntura. Las evaluaciones se llevaron a cabo antes, después de finalizar el tratamiento y siete días después (seguimiento), a través del Inventario de Ansiedad Estado y medición de frecuencia cardíaca y respiratoria. Se utilizó las Ecuaciones de estimación generalizadas.

**Resultados:**

Hubo una reducción de los niveles de ansiedad en ambos grupos, entre la evaluación inicial y final, y entre la inicial y el seguimiento. No hubo diferencias entre los grupos en las evaluaciones final y de seguimiento. La frecuencia cardíaca se redujo en el grupo Aguja entre las evaluaciones final y de seguimiento*,* e inicial y de seguimiento; el grupo Láser se mantuvo constante a lo largo del tiempo y los grupos no difirieron entre las evaluaciones final y de seguimiento. No hubo cambios con el tiempo en la frecuencia respiratoria en ambos grupos y no difirieron en las evaluaciones finales y de seguimiento. Hubo mayor aparición de reacciones adversas en el grupo que recibió la intervención con agujas, a pesar de la leve intensidad.

**Conclusión:**

Ambas intervenciones fueron efectivas para reducir el nivel de ansiedad en estudiantes universitarios. Registro Brasileño de Ensayos Clínicos RBR-8cxnvr2.

## INTRODUCTION

Covid-19 has triggered a serious crisis in people’s mental health^(1)^. Emotional disorders affected young people, adults and, mainly, university students^(2,3)^, anxiety being the main one^(4)^. In the university context, anxiety is present at significant levels and prevalence even in the post-pandemic context^(5,6)^, which can significantly affect the academic performance and mental health of this population^(2,7)^.

In this scenario, ear acupuncture stands out as an effective intervention for the health care of individuals with emotional disorders^(8)^. This is one of the 29 Integrative and Complementary Health Practices (*PICS*) institutionalized in the Brazilian Public Health System (*SUS*), which are seen as important health care strategies, especially when considering the integrality of the human being and its complementary nature to conventional treatments, and provide support and qualified listening^(9)^. Several professionals are qualified to use *PICS*, including nurses, according to resolution of the Federal Nursing Council no. 739/2024^(10)^, which recommends training for ear acupuncture through free courses, with a minimum workload of 80 hours.

This intervention has its origins in the precepts of Traditional Chinese Medicine, and is based on the stimulation of specific points in the ear, which triggers modulations in the Central Nervous System, to reestablish homeostasis^(11)^. Furthermore, it has the advantages of having minimal side effects and being easy to operate^(7,12)^.

Needles are the most common devices for applying ear acupuncture^(13,14)^, being considered the gold standard for the intervention. With technological evolution and the aim of making the techinque application safer for both clients and professionals, new application devices are gaining prominence, such as the low-intensity laser^(14)^. When used at low light intensity, the minimum light energy promotes the activation of biologically reactive zones in the ear (the auricular acupoints) and, consequently, the reestablishment of the body’s homeostasis^(15)^. Thus, the reduction in invasiveness, compared to intervention using needles, constitutes a significant factor that favors the acceptance of treatment using laser^(16)^.

In this context, to minimize risks and promote therapeutic care as effective as that of ear acupuncture with needles, in addition to addressing the potential of laser therapy and the scarcity of studies using laser, especially for the treatment of emotional disorders in university students, this study proposed to compare the effectiveness of ear acupuncture with laser and needles in the treatment of anxiety in university students in the post-pandemic context of Covid-19, as well as to evaluate the possible symptoms or adverse reactions triggered by the interventions.

## METHOD

### Design of Study

Randomized, multicenter, parallel, open clinical trial, consisting of two groups: ear acupuncture with needles (needle group - NG - control) and ear acupuncture with laser (laser group - LG - experimental), reported according to recommendations of *Consolidated Standards of Reporting Trials* (CONSORT)^(17)^.

### Local

The study was carried out at two public universities in the state of Minas Gerais, between December 2023 and April 2024.

### Population and Selection Criteria

The study population consisted of 1,093 university students from all undergraduate courses at the aforementioned universities, from four areas of knowledge: agricultural sciences; biological and health sciences; exact and technological sciences; human sciences, literature and arts, with 62 courses from one university, distributed across three *campi*, and 40 of the other, also in three *campi,* with moderate or high levels of anxiety (i.e., a score greater than nine on the *Depression, Anxiety and Stress Scale* (DASS-21)^(18)^. The study was released on the universities’ official communication channels, in institutional emails, and through the distribution of pamphlets. Students aged 18 or over and with time available to complete assessments and treatment sessions were included in the study. The following were excluded: those who did not accept being randomly allocated to the two study groups; who presented rejection or fear of receiving the interventions, history of allergy to micropore tape or metal, history of photosensitivity and skin cancer in the head and neck region, compromised immune system, epilepsy; who used chemical peeling or isotretinoin up to six months prior to the start of treatment; ear piercings close to the selected ear points; who used hearing aids or cardiac pacemaker; who had tattoos, injuries, inflammation or deformities in the ear; who were pregnant or in the lactation period; women who planned to become pregnant during the study period; and those who received any PICS in the last three months.

The following discontinuation criteria were also considered: absence from any of the assessments; loss of two consecutive ear acupuncture sessions or absence of more than 10 days between sessions; presentation of an inflammatory reaction in the ear; intense discomfort at the site of application of the needles or laser; or expression of the desire to discontinue participation in the research.

### Sample Size Calculation and Randomization

Sample calculation was based on the difference in means within the groups between the initial ( $\bar{\text{X}}$ = 2.71; σ^2^ = 9.22) and final ( $\bar{\text{X}}$ = 1.47; σ^2^ = 11.29) assessments, in relation to the level of anxiety, which was assessed using the State Anxiety Inventory (STAI-S)^(19)^. The *software* RMASS2® was used: *Repeated Measurements with Attrition: Sample Sizes for 2 Groups*, after carrying out a pilot test with 20 individuals (10 per group). For a significance level of 5%, power of 90%, and medium effect size (0.5), a minimum sample of 40 individuals was estimated in each group. To guarantee the sample size, the initial calculation was corrected by 30%, which increased the sample to 52 individuals per group.

The randomization of the groups was performed by an external researcher, in a 1:1 ratio, in a block of 10 students. For each block, a sequence of random numbers was generated through the site (http://www.randomization.com/). After generating the list, the sequence of groups was cut and placed in an opaque, numbered, and sealed envelope. Immediately before the first AA session, the envelope with the randomization was opened by the interventionist and the students were randomly allocated to the control group (ear acupuncture with needles – NG; n = 61) or to the experimental group (ear acupuncture with laser – LG; n = 65).

Study participants and evaluators were not masked, due to the peculiarities of the interventions applied; that is, both could identify the allocation of groups by the presence or absence of semi-permanent needles in the auricle in the NG.

### Interventions and Data Collection

Interventions were applied by nurse researchers, with two to 12 years of experience in the application of ear acupuncture.

The sessions were carried out with a one-week interval, changing the pinna at each session, totaling five sessions. The auricular map recommended by the *World Federation of Acupuncture-Moxibustion Societies* ^(20)^ and an electric acupoint finder (EL30 Finder NKL Basic® with red differential pen) for precise location of auricular points were used. Nine previously validated auricular points were applied^(21)^: *Shenmen* (TF_4_), Sympathetic Nervous System (AH_6a_), Kidney (CO_10_), Liver (CO_12_), Spleen (CO_13_), Brainstem (ATS_3,4i_), Lung 1 (CO_14_), Heart (CO_15_) and *Yang* of Liver 2 (HX_8_ – lower portion).

Pinna antisepsis was performed with cotton and 70% ethyl alcohol. In the NG, ear acupuncture was performed with semi-permanent, sterilized and disposable needles, size 0.20 × 1.5 millimeters (Complementar Agulhas®). In the LG, a low-intensity diode laser was used, with a wavelength of 808 nanometers (infrared), an average power of 100 mW and a beam spot area of 1 cm^2^ (ACP Therapy - DCM®). The laser was applied in contact with the skin, continuously, on a single point (perpendicular to the skin), and in a stationary way. The application time was 40 seconds for each stimulated point, with an energy dose of 4 joules^(22)^ (irradiance per point: 100 mW/cm^2^; fluence per point: 4.0 J/cm^2^). A spacer (area: 0.09842 cm^2^; diameter: 3.54 mm - DMC®) was used at the tip of the laser cannula, with the aim of maintaining a safe distance between the cannula and the participant’s skin. Before the laser application, a film of polyvinyl chloride was placed on the spacer and in the equipment cannula for greater hygiene, being changed after each session. Furthermore, the volunteer and therapist used specific protective glasses to avoid possible damage to the retina from the laser light.

Participants were assessed at three times: 1- Initial assessment (before the first AA session: students responded, online, the Sociodemographic and Psychosocial Characterization Instrument and the STAI-S^(19)^, and the heart rate and respiratory rate parameters were measured by trained examiners. 2- Final evaluation (one week after the fifth session): the participants responded, online, to STAI-S^(19)^ and the satisfaction and adverse reactions questionnaire, and heart and respiratory rates were measured by the same trained examiners. 3- Follow up Assessment (in a seven-day follow-up period): participants responded, online, to STAI-S^(19)^ and the heart and respiratory rates were reassessed again by the same examiners.

For the sociodemographic characterization, the following were investigated: age; sex; self-declared skin color; marital status; housing; work activity; and family income. The clinical and psychosocial profile was established using the following variables: positive diagnosis of Covid-19; follow-up with a psychiatrist and/or psychologist; clinical diagnosis related to mental health; time of diagnosis; use of psychotropic drugs and time of start of use; and self-assessment of physical and mental health (on a scale of zero to 10). In addition, to investigate the academic profile, the course area, student assistance, grant of scholarships, and the influence of the Covid-19 pandemic on academic performance were assessed, aiming future professional prospects and the overload of academic activities.

Anxiety levels were also investigated using STAI-S^(19)^ (primary outcome), as well as heart rate and respiratory rate parameters (secondary outcomes).

STAI-S is a self-report questionnaire that assesses the anxiety status (momentary emotional state)^(19)^. It consists of twenty statements, with four answer options (1 - absolutely not to 4 - very much)^(19)^. The score ranges from 20 to 80 points, with low anxiety level (20–30), medium anxiety level (31–49), and high anxiety level (≥ 50)^(23)^. For this evaluation, a version *online* of the questionnaire, via platform *Google Forms®*, was generated, which was answered in the form of a self-report.

To assess heart and respiratory rates, the participant remained at rest for 20 minutes, sitting on a chair, with his back supported. Heart rate was assessed with a portable digital oximeter (Oxiled/LED G-TECH®) positioned on the index finger of the participant’s left hand, for one minute, while the participant’s respiratory rate was measured through the observation of his/her chest or abdomen, considering the number of times the combined movements of inhalation and exhalation were performed in one minute, without the individual realizing the intention of the evaluator, who used a stopwatch.

To assess the level of satisfaction with the treatment, a Likert-type scale, from one to five points (1 - extremely dissatisfied to 5 - extremely satisfied) was applied; possible adverse reactions resulting from the interventions were graded from zero (no discomfort) to 10 (unbearable discomfort).

### Data Analysis and Treatment

The collected data were grouped into a database, using the Microsoft Office Excel (2021) application and analyzed with the statistical software *Statistical Package for the Social Sciences* (SPSS), version 26.0, by a researcher masked to group allocation.

Homogeneity between the two groups was verified using the Mann-Whitney and Chi-Square tests, at 5% significance, in relation to sociodemographic, clinical, psychosocial, and academic variables.

Generalized Estimating Equation (GEE) models were used to estimate and test the effect of group allocation, adjusting for the effect of time (initial, final and follow up assessments) and interaction (group * time), in the outcome variables. The variables were treated assuming gamma distribution and logarithmic link function. The model with the smallest *Quasi Likelihood Under Independence Model Criterion* (QIC) and the best fit was considered as the final (unstructured) model. Statistical significance was set at p < 0.05. The Bonferroni post-hoc test was used to compare the means of significant correlations between predictors and outcomes.

### Ethical Aspects

The study was approved by the Research Ethics Committee, with opinion number 5.700.107/CAAE: 63318222.0.0000.5153. All students who agreed to participate in the study signed the Free and Informed Consent Form in digital format. At the end of the study, all participants were invited to complete the same treatment sessions as the group to which they were not initially allocated.

## RESULTS

**Figure 1:**
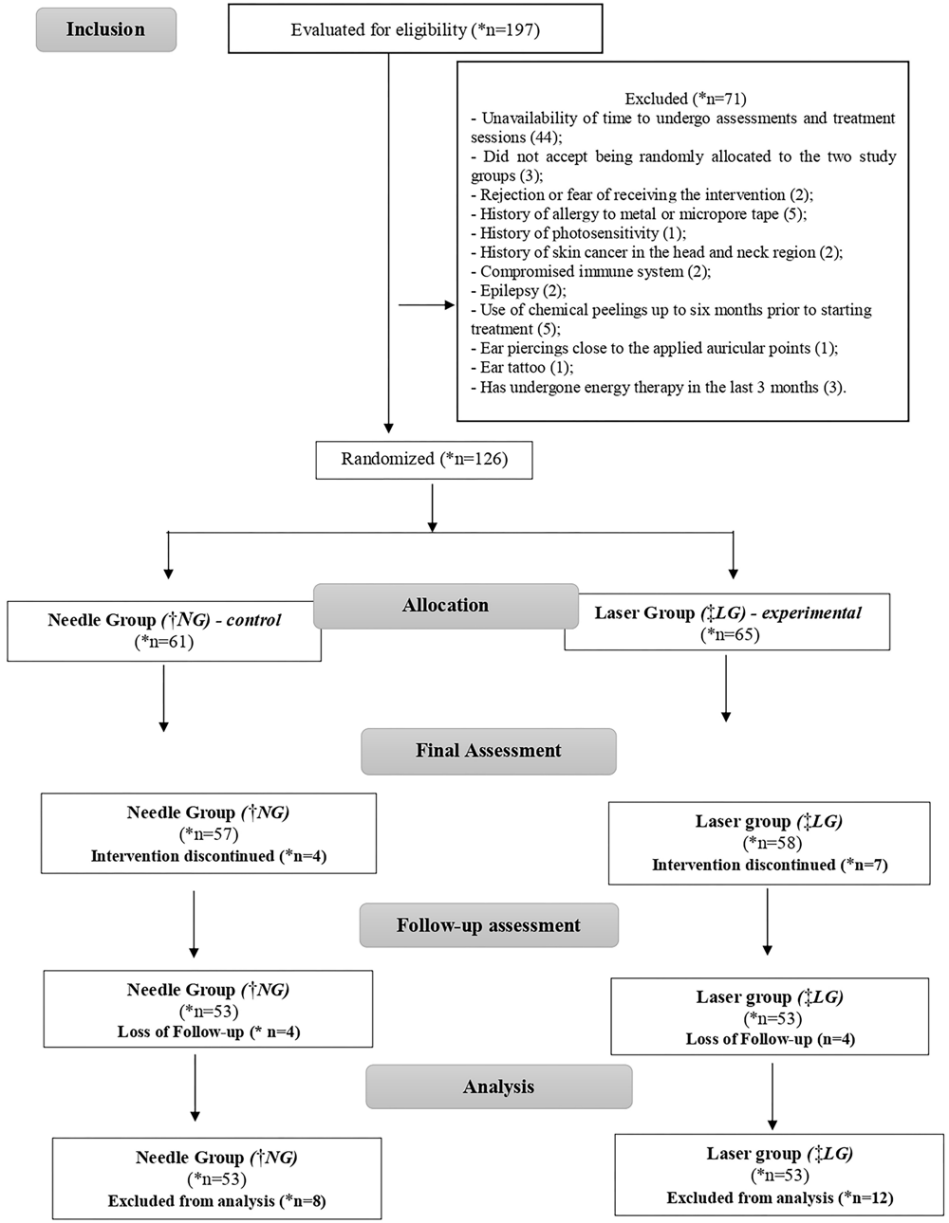
Sample tracking flowchart.

Source: Adapted according to the Consort model^(17)^.

Note: *n = absolute frequency; ^†^ NG = Needle Group (control); ^‡^LG = Laser Group (intervention).

Initially, 197 students were assessed for eligibility and 71 did not meet the inclusion criteria. The eligible sample obtained consisted of 126 students, who were considered for randomization (61 in NG and 65 in LG) and 106 completed the study. There was a loss of 20 students (15.85%) throughout the study, who were excluded from the statistical analysis once the sample size was reached (Figure 1).

Data in Table 1 show the comparison between the groups according to the sociodemographic, clinical, psychosocial, and academic profile. There were no statistically significant differences between the groups in relation to these variables, which points to homogeneity within them.

**Table 1:** Characterization of the sample regarding sociodemographic, clinical, psychosocial and academic profile – Viçosa, MG, Brazil, 2024. (n = 106).

Sociodemographic profile				
Variables		*NG (n = 53)	^†^ LG (n = 53)	p value
Age (^‡^ $\bar{\text{X}}$ ± ^§^sd)	Years	22.83 ± 4.13	22.04 ± 4.98	0,700**
Sex	Male	16 (30.2)	10 (18.9)	0,176^^††^^
^||^n (^¶^%)	Female	37 (69.8)	43 (81.1)	
Self-declared skin color	White	30 (56.6)	32 (60.4)	0,764^^††^^
^||^n (^¶^%)	Yellow	0 (0.0)	0 (0.0)	
	Brown	16 (30.2)	16 (30.2)	
	Black	6 (11.3)	5 (9.4)	
	Indigenous	1 (1.9)	0 (0.0)	
Marital status	Single	45 (84.9)	44 (83.0)	0.603^^††^^
^||^n (^¶^%)	Married/Common law marriage	8 (15.1)	8 (15.1)	
	Widower	0 (0.0)	1 (1.9)	
	Divorced	0 (0.0)	0 (0.0)	
Housing	Alone	7 (13.2)	9 (17.0)	0.599^^††^^
^||^n (^¶^%)	With family	18 (34.0)	13 (24.5)	
	Republic	24 (45.3)	24 (45.3)	
	Student accommodation	4 (7.5)	7 (13.2)	
Work activity	No	47 (88.7)	46 (86.8)	0,767^^††^^
^||^n (^¶^%)	Yes	6 (11.3)	7 (13.2)	
Family income	1 to 1 and ½ minimum wages	16 (30.2)	15 (28.3)	0.465^^††^^
^||^n (^¶^%)	2 to 3 minimum wages	22 (41.5)	19 (35.8)	
	4 to 5 minimum wages	11 (20.8)	12 (22.6)	
	6 or more minimum wages	4 (7.5)	7 (13.2)	
**Clinical and psychosocial profile**				
Positive diagnosis for Covid-19	No	30 (56.6)	30 (56.6)	1.000^^††^^
^||^n (^¶^%)	Yes	23 (43.4)	23 (43.4)	
Follow-up with a psychiatrist	No	34 (64.2)	33 (62.3)	0.884^^††^^
^||^n (^¶^%)	Already did it before the pandemic	7 (13.2)	6 (11.3)	
	Started doing it during/after the pandemic	12 (22.6)	14 (26.4)	0.495^^††^^
Follow-up with a psychologist	No	27 (50.9)	26 (49.1)	0.455^^††^^
^||^n (^¶^%)	Already did it before the pandemic	12 (22.6)	8 (15.1)	
	Started doing it during/after the pandemic	14 (26.4)	19 (35.8)	0.150^^††^^
Clinical diagnoses related to mental health	Depression	10 (18.9)	9 (17.0)	0.800^^††^^
^||^n (^¶^%)	Anxiety	27 (50.9)	21 (39.6)	0.242^^††^^
	Post Traumatic Stress Syndrome	0 (0.0)	1 (1.9)	0.315^^††^^
	Panic syndrome	2 (3.8)	0 (0.0)	0.153^^††^^
	Mood disorders	1 (1.9)	2 (3.8)	0.558^^††^^
	Obsessive Compulsive Disorder	0 (0.0)	2 (3.8)	0.153^^††^^
	Attention deficit hyperactivity disorder	1 (1.9)	1 (1.9)	1.000^^††^^
	Personality disorders	0 (0.0)	1 (1.9)	0.315^^††^^
	Fobia social	1 (1.9)	1 (1.9)	1.000^^††^^
	Sleep disorders/disturbances	3 (5.7)	3 (5.7)	1.000^^††^^
Timing of clinical diagnosis related to mental health	Does not have any diagnosis	18 (34.0)	22 (41.5)	0.638^^††^^
^||^n (^¶^%)	I already had the diagnosis before the pandemic	18 (34.0)	14 (26.4)	
	Established during the pandemic	17 (32.1)	17 (32.1)	
Use of psychotropic drugs	Antidepressant	19 (38.8)	18 (34.0)	0.839^^††^^
^||^n (^¶^%)	Anxiolytic	19 (38.8)	17 (32.1)	0.682^^††^^
	Antipsychotic	1 (1.9)	3 (5.7)	0.308^^††^^
	Mood stabilizer	1 (1.9)	3 (5.7)	0.308^^††^^
	Non-benzodiazepine hypnotic	1 (1.9)	0 (0.0)	0.315^^††^^
	Amphetamines	1 (1.9)	0 (0.0)	0.315^^††^^
Time of initiation of psychotropic drug use	Does not use	30 (56.6)	30 (56.6)	0.957^^††^^
^||^n (^¶^%)	Before the pandemic	11 (20.8)	10 (18.9)	
	After the pandemic	12 (22.6)	13 (24.5)	
Physical health Self-assessment (^‡^ $\bar{\text{X}}$ ^§^sd)	zero to 10	5.34 ± 2.12	5.71 ± 2.04	0.467**
Mental health self-assessment (^‡^ $\bar{\text{X}}$ ^§^sd)	zero to 10	4.33 ± 1.63	4.45 ± 1.74	0.819**
**Academic profile**				
Course area	Agricultural Sciences	9 (17.0)	10 (18.9)	0,982^^††^^
^||^n (^¶^%)	Biological and Health Sciences	23 (43.4)	22 (41.5)	
	Exact and Technological Sciences	9 (17.0)	10 (18.9)	
	Human Sciences, Arts and Letters	12 (22.6)	11 (20.8)	
Receive student assistance	No	38 (71.7)	32 (60.4)	0.218^^††^^
^||^n (^¶^%)	Yes	15 (28.3)	21 (39.6)	
Receives a scholarship for teaching, research, or extension	No	40 (75.5)	41 (77.4)	0.819^^††^^
^||^n (^¶^%)	Yes	13 (24.5)	12 (22.6)	
Academic performance	No change	9 (17.0)	4 (7.5)	0,171^^††^^
^||^n (^¶^%)	Improved	3 (5.7)	7 (13,2)	
	Got worse	41 (77.4)	42 (79.2)	
Future professional perspective	No change	16 (30.2)	13 (24.5)	0,258^^††^^
^||^n (^¶^%)	Improved	5 (9.4)	11 (20.8)	
	Got worse	32 (60.4)	29 (54,7)	
Overload of academic activities after the Covid-19 pandemic	No	5 (9.4)	5 (9.4)	1,000^^††^^
^||^n (^¶^%)	Yes	48 (90,6)	48 (90,6)	

Note:

*NG = Needle Group (control)

^^†^^LG = Laser Group (experimental)

^‡^ $\bar{\text{X}}$ = sample mean

^§^sd = standard deviation; ^||^n = absolute frequency

^¶^% = relative frequency

** = Mann-Whitney

^^††^^ = Pearson’s Chi-square.

Table 2 presents the anxiety levels in the sample studied. Initially, the groups were homogeneous in relation to this variable, since there was no statistically significant difference between them. A statistically significant reduction was observed in the NG and LG, between the initial and final evaluations (mean difference: 9.34 and 7.83 respectively), and initial and follow up (mean difference: 7.81 and 6.53, respectively). The level of anxiety remained constant between the final and follow-up assessments in both groups, that is, the reduction achieved at the end of the treatment remained for seven days after its end. There was no statistically significant difference between the groups in the final and follow up assessments, which confirms that the application of ear acupuncture with needles or laser has the same effect.

**Table 2:** Analysis of anxiety level, expressed as mean, standard error and 95% confidence interval – Viçosa, MG, Brazil, 2024. (n = 106).

Anxiety	Initial	Final	Follow up	p value CI 95%		
	^†^ $\bar{\text{X}}$ (standard error)	^†^ $\bar{\text{X}}$ (standard error)	^†^ $\bar{\text{X}}$ (standard error)	Initial–Final	Final–Follow up	Initial–Follow up
^‡^NG (^§^n = 53)	58.85 (1,215)	49.51 (1,436)	51.04 (1,942)	**<0,001** **5.93; 12.74**	0,801-4.83; 1.77	**<0,001** **3.76; 11.86**
^||^LG (^§^n = 53)	57.87 (1,193)	50.04 (1,408)	51.34 (1,435)	**<0,001** **4.56; 11.10**	0,921-4.35; 1.75	**<0,001** **2.87; 10.19**
p value	1.000	1.000	1.000			
*CI 95%	-4.02;5.98**	-6.43;5.38**	-7.39;6.79**			

Note:

*95% CI = 95% confidence interval for the difference in means

^^†^^ $\bar{\text{X}}$ = sample mean

^‡^NG = Needle Group (control)

^§^n = absolute frequency

^||^LG = Laser Group (experimental)

** = Difference in means: NG – LG.

Table 3 presents data on heart and respiratory rates of study participants. Initially, the groups were homogeneous in relation to these two variables. Regarding heart rate, a statistically significant reduction was observed in the NG between the final and follow-up (mean difference: 3.94) assessments and between the initial and follow-up assessments (mean difference: 4.38). Meanwhile, the LG remained constant over time. Furthermore, the groups did not differ from each other in the final and follow-up assessments, demonstrating the same effects exerted by the needle and laser applications.

**Table 3:** Analysis of heart and respiratory rates, expressed as mean, standard error and 95% confidence interval – Viçosa, MG, Brazil, 2024. (n = 106).

	Frequencies	Initial	Final	Follow up	p value *CI 95%		
		^†^ $\bar{\text{X}}$ (standard error)	^†^ $\bar{\text{X}}$ (standard error)	^†^ $\bar{\text{X}}$ (standard error)	Initial–Final	Final–Follow up	Initial–Follow up
**Cardiac**	^‡^NG (^§^n = 53)	83.91 (1.954)	83.47 (1.517)	79.53 (1.496)	1.000 -3.80; 4.67	**0.022** **-0.33; 8.42**	**0.029** **-0.42; 7.47**
	^||^LG (^§^n = 53)	81.62 (1.552)	83.09 (1.263)	83.40 (1.418)	1.000-5.61; 2.67	1.000-3.62; 3.02	0.912-5.90; 2.36
	p value	1.000	1.000	0.908			
	*CI 95%	-5.04; 9.91**	-5.42; 6.17**	-9.92; 2.18**			
**Respiratory**	^‡^NG (^§^n = 53)	16.77 (0.440)	16.47 (0.409)	15.60 (0.440)	1.000 -0.84; 1.45	0.168 -0.22; 1.96	0.054 -0.01; 2.35
	^||^LG (^§^n = 53)	15.79 (0.358)	15.68 (0.312)	15.68 (0.418)	1.000 -0.78; 1.01	1.000 -0.89; 0.89	1.000 -1.01; 1.23
	p value	1.000	1.000	1.000			
	*CI 95%	-0.68; 2.65**	-0.72; 2.30**	-1.86; 1.71**			

Note:

*95% CI = 95% confidence interval for the difference in means

^^†^^ $\bar{\text{X}}$ = sample mean

^‡^NG = Needle Group (control)

^§^n = absolute frequency

^||^LG = Laser Group (experimental)

** = Difference in means: NG – LG.

Regarding respiratory rate, there were no statistically significant changes over time in either group. Furthermore, the groups did not differ statistically from each other in the final and follow-up assessment, confirming the equality of effect between the interventions (Table 3).

Regarding participants’ level of satisfaction in relation to the treatment performed, it is noteworthy that 39.6% (n = 21) of participants in the NG and 41.5% (n = 22) in LG were extremely satisfied; 41.5% (n = 22) in the NG and 37.7% (n = 20) in LG were satisfied; 15.1% (n = 8) in NG and 17.0% (n = 9) in LG were not sure; no participant in NG and 1.9% (n = 1) in LG were dissatisfied; and 3.8% (n = 2) and 1.9% (n = 1) were extremely dissatisfied, respectively, with a p-value = 0.825 according to Pearson’s Chi-square test.

Table 4 shows the frequency and intensity of symptoms or adverse reactions. Pain due to the needles remaining in the ear, hyperemia, itching, edema, and scaling were more frequent in the NG. Regarding the intensity of symptoms, only headache and ear redness did not differ between the groups; the others were more intense in the NG compared to LG, but classified as mild.

**Table 4:** Frequency and intensity of symptoms or adverse reactions of study participants who received ear acupuncture with needles and laser – Viçosa, MG, Brazil, 2024. (n = 106).

Symptom or adverse reaction	Frequency *n (%)		p value
	^‡^NG (*n = 53)	^§^LG (*n = 53)	
Ear pain	46 (86.8)	06 (11.3)	<0.001**
Ear Hyperemia	31 (58.5)	10 (18.9)	<0.001**
Itchy ear	29 (54.7)	08 (15.1)	<0.001**
Intervention-related headache	19 (35.8)	17 (32.1)	0.682**
Ear edema	16 (30.2)	00 (0.00)	<0.001^^††^^
Ear scaling	12 (22.6)	03 (5.70)	0.023^^††^^
Enlarged lymph nodes	08 (15.1)	02 (3.80)	0.093^^††^^
Ear Redness	00 (0.00)	01 (1.90)	1.000^^††^^
**Intensity (scale from zero to 10) ^|^ $\bar{\text{X}}$ (^¶^dp)**			
Ear pain	3.23 (2.44)	0.19 (0.65)	**<0.001^^††^^**
Ear Hyperemia	1.89 (2.18)	0.23 (0.50)	**<0.001^^††^^**
Itchy ear	2.28 (2.77)	0.28 (0.74)	**<0.001^^††^^**
Intervention-related headache	1.30 (2.39)	1.11 (1.91)	0.761^^††^^
Ear edema	0.74 (1.51)	0.00 (0.00)	**<0.001^^††^^**
Ear scaling	0.66 (1.55)	0.11 (0.50)	**0.012^^††^^**
Enlarged lymph nodes	0.55 (1.46)	0.13 (0.73)	**0.048^^††^^**
Ear Redness	0.00 (0.00)	0.02 (0.14)	0.317^^††^^

Note:

*n = absolute frequency

^^†^^% = relative frequency

^‡^NG = Needle Group (control)

^§^LG = Laser Group (experimental)

^||^ $\bar{\text{X}}$ = sample mean

^¶^sd = standard deviation

** = Pearson’s chi-square

^^††^^ = Fisher’s exact

^‡‡^ = Mann-Whitney.

## DISCUSSION

The results of this study demonstrated that ear acupuncture performed with laser has similar effectiveness to ear acupuncture performed with semi-permanent needles for the treatment of anxiety in university students in the post-COVID-19 pandemic context. Furthermore, there was a greater occurrence of symptoms or adverse reactions in the group receiving the intervention with needles, despite the mild intensity.

The use of needles in ear acupuncture is already consolidated in scientific circles. In fact, scientific evidence^(21,24)^ already point to the effectiveness of needle intervention in the treatment of emotional disorders. In fact, these are the most traditional acupuncture devices with proven effectiveness. Regarding the use of laser in emotional disorders, scientific evidence is still incipient and there is a diversity of dosimetric parameters adopted, which represents an area that requires further investigation.

To date, the use of laser for anxiety^(25)^, sleep^(25)^, temporomandibular disorders^(25)^, and depression associated with this condition^(22)^ stands out. Fernandes and contributors^(25)^ performed 10 weekly sessions, with low-intensity infrared laser, wavelength of 808 nm (infrared), with a power of 100 mW, on the auricular points *Shenmen*, Upper Limb, Zero, Stomach, Maxilla, Mandible, Anxiety, and Stress, and found that ear therapy was effective in treating anxiety; however, it was not effective in the results of sleep disorder and symptoms of temporomandibular dysfunction. Rodrigues and contributors^(22)^, on turn, performed the application of the laser on the points *Shenmen*, Temporomandibular joint and Heart, once a week, for eight sessions. The laser used was a 75 W pulsed diode laser (InGaAs\GaAs), output power of 50 mW, wavelength of 904 nm (infrared), at 4 J/cm^2^ for 24 seconds at each irradiated auricular point^(22)^. The authors found that low-level laser ear therapy improved the physical and emotional symptoms of temporomandibular dysfunction, with similar results to the group that used occlusal splints^(22)^. Given the above, it is clear that this is an area that still requires research, since there is little scientific evidence on the subject, there is no standardization regarding dosimetric parameters, and the results are still divergent between studies.

In this context of health care for individuals with emotional disorders, the present study contributes with further scientific evidence on the effectiveness of laser, which reinforces its use as a non-pharmacological strategy for relieving anxiety. Thus, the implementation of this therapy in the university environment may provide students with access to this effective *PICS*, carried out through a safe and easy-to-use technological resource, but which is not yet fully available in the *SUS* healthcare networks. This may contribute to promoting students’ mental health and, consequently, favor better academic performance and a more positive professional future perspective.

It is also worth noting that *PICS* constitute important care strategies, by focusing attention on the user and their demands, providing expanded, resolute, and comprehensive assistance^(26)^. In the context of nursing^(10)^, the nurse can implement ear acupuncture, with needle or laser, in their clinical practice, as an effective intervention for the treatment and prevention of anxiety, which is a nursing problem under their responsibility.

In addition to anxiety reduction, the present study also evaluated other parameters, such as heart and respiratory rates. Despite a reduction in heart rate among participants undergoing ear acupuncture with needles, when comparing both interventions, there was no statistically significant difference between them at the end of treatment.

These findings related to heart rate are in line with a study that evaluated the effects of ear acupuncture with a needle in the vagus nerve over the cardiac autonomic nervous system^(27)^. This study suggests that stimulation performed at points located in the cymba and cavum conchae may have triggered stimulation of the vagus nerve, thus generating changes in heart rate^(28)^. Furthermore, because the needles promote pain and a local microinflammatory response^(29)^, the points remain reactive, which makes changes more noticeable in the needle group.

In contrast, in the present study there were no statistically significant changes in respiratory rate in either group. This finding contrasts with that of a clinical trial aimed at evaluating the effect of ear therapy on anxiety and physiological parameters of patients undergoing coronary angiography^(30)^. The intervention was applied through light acupressure, at the Shenmen, Relaxation, Tranquilizing and Endocrine acupoints on the non-dominant side, each of which was stimulated with the thumb for one minute in a counterclockwise direction, 60 minutes before the angiography. Respiratory rate was assessed by observing chest movements by the same researcher for one minute^(30)^. It is suggested that the effects on respiratory rate differed from those observed in the present study due to the use of other auricular points, such as Endocrine, Relaxation and Tranquilizing^(30)^ ones, which also have an effect on anxiety.

Regarding symptoms or adverse reactions, ear pain was the most common and intense symptom reported by participants in the needle group. However, the severity was considered to be of low intensity. This indicates that, despite adverse effects, students generally tolerate ear acupuncture with needles well^(7,12)^, since the reactions were not severe.

In contrast, the use of laser has proven to be advantageous considering that the results are comparable to the use of needles in relation to anxiety levels, which is the gold standard, and presented less intense reactions. It should also be noted that the laser is a non-invasive device, which does not present a risk of infection, is painless, easy and quick to apply and, above all, beneficial for people with a phobia of needles^(14,15)^. It is important to highlight that adverse reactions very rarely occur in ear acupuncture. Fatal reactions or irreversible adverse events have not been documented in the scientific literature to date. Minor adverse events, such as local pain, bleeding, or infections may occur after the use of needles, which are preventable when correct techniques for disinfecting materials, antisepsis and constant inspection of the ear are adopted^(14)^. It should also be noted that, as far as we know, the scientific literature does not yet present data comparing laser to needle in relation to the infection potential of the interventions, which highlights the innovative nature of the present study. Regarding the participants’ satisfaction with the interventions received, both groups were satisfied. This confirms the safety and effectiveness of the technique.

As limitations of this study, we first highlight the lack of masking of participants, which was impossible due to the peculiarities of the interventions applied. Despite this, the interventionists were different from the evaluators to avoid any interference in the data collected, and the professional who performed the analysis of the results was unaware of the group allocation. Secondly, sample loss stands out. However, this was not significant and the number of students reached the value estimated by the sample calculation, which confirms the magnitude of the findings. Another factor that deserves to be emphasized is the assessment of the respiratory rate outcome through observation, since it may have been subject to interference by the evaluator.

Despite the limitations, the effectiveness of ear acupuncture with needles and laser demonstrated in this study contributes to strengthen the growing evidence base supporting the integration of this intervention as a complementary therapeutic option in the treatment of anxiety, which can be adopted by nurses in their clinical practice, to expand their scope of action. Furthermore, the use of ear acupuncture, both with needles and laser, is encouraged for the management of anxiety in university students by higher education institutions, to possibly have an impact on academic performance and quality of life, with possible impacts on reducing the consumption of psychotropic drugs, which can cause dependence, and the abuse of alcohol and other drugs. For future research, studies with larger samples are recommended to elucidate the effects of ear acupuncture with both devices on vital parameters that may be altered due to the presence of anxiety, in addition to other outcomes that may be associated with anxiety, such as impaired mood regulation, chronic sadness, risk of suicidal behavior, stress overload, among others, which are also the responsibility of the nurse.

## CONCLUSION

Ear acupuncture, both with needles and laser, was effective in reducing anxiety levels in university students, with effects observed seven days after the end of treatment. Nevertheless, there was no statistically significant difference between the groups in terms of heart rate and respiratory rate parameters, despite the reduction in heart rate over time in the group treated with needles. Moreover, it was shown that the use of laser in ear acupuncture is advantageous when considering the adverse reactions presented by the needles.

## Data Availability

https://doi.org/10.48331/scielodata.DX72SY
