# Supplementary material for: Ear acupuncture with laser and needles in the treatment of anxiety in university students: a randomized clinical trial
Source: Rev Esc Enferm USP. 2025 Jan 27;58:e20240239. doi: 10.1590/1980-220X-REEUSP-2024-0239en (PMC11773684; doi:10.1590/1980-220X-REEUSP-2024-0239en)
Supplement: Translation

# Translation: Auriculoacupuntura com laser e com agulhas no tratamento da ansiedade de universitários: ensaio clínico randomizado*

**DOI:** 10.1590/1980-220X-REEUSP-2024-0239pt

**Authors:** Ana Paula da Silva Lemos, Bárbara Guimarães Lourenço, Érika de Cássia Lopes Chaves, Luana Vieira Toledo, Tânia Couto Machado Chianca, Caroline de Castro Moura

**Affiliations:** 1 Universidade Federal de Viçosa, Programa de Pós-graduação em Ciências da Saúde, Viçosa, MG, Brasil.; 2 Universidade Federal de Viçosa, Departamento de Medicina e Enfermagem, Viçosa, MG, Brasil.; 3 Universidade Federal de Alfenas, Escola de Enfermagem, Alfenas, MG, Brasil.; 4 Universidade Federal de Minas Gerais, Departamento de Enfermagem Básica, Belo Horizonte, MG, Brasil.

**Keywords:** Acupuncture, Ear, Low-Level Light Therapy, Universities, Ansiedade, COVID-19.

## Abstract

**Objetivo:**

Comparar a efetividade da auriculoacupuntura com laser e agulhas no tratamento da ansiedade em universitários no contexto pós pandêmico da Covid-19, bem como avaliar os possíveis sintomas ou reações adversas desencadeados pelas intervenções.

**Método:**

Ensaio clínico randomizado realizado com 126 universitários, alocados nos grupos “Agulha” (controle) e “Laser” (experimental). Foram realizadas cinco sessões de auriculoacupuntura. As avaliações foram realizadas antes, após o término do tratamento e sete dias após (*follow up)*, por meio do Inventário de Ansiedade Estado e da mensuração das frequências cardíaca e respiratória. Empregou-se o *Generalized Estimating Equations*.

**Resultados:**

Houve redução nos níveis de ansiedade em ambos os grupos, entre as avaliações inicial e final, e inicial e *follow up*. Não houve diferença entre os grupos nas avalições final e *follow up*. A frequência cardíaca reduziu no grupo Agulha entre as avaliações final e *follow up*, e inicial e *follow up*; o grupo Laser permaneceu constante ao longo do tempo e os grupos não diferiram entre si entre o final e *follow up*. Não houve alteração ao longo do tempo na frequência respiratória em ambos os grupos e eles não diferiram entre si nas avaliações final e *follow up*. Houve maior ocorrência de reações adversas no grupo que recebeu a intervenção com agulhas, apesar da intensidade leve.

**Conclusão:**

Ambas as intervenções foram efetivas para redução do nível de ansiedade em universitários. Registro Brasileiro de Ensaios Clínicos RBR-8cxnvr2.

## INTRODUÇÃO

A Covid-19 desencadeou grave crise na saúde mental das pessoas^(1)^. As desordens emocionais acometeram jovens, adultos e, principalmente, universitários^(2 ,3)^, sendo a ansiedade a principal delas^(4)^. No contexto universitário, a ansiedade faz-se presente em níveis e prevalências expressivas ainda no contexto pós pandemia^(5 ,6)^, o que pode afetar significativamente o desempenho acadêmico e a saúde mental dessa população^(2 ,7)^.

Destaca-se, nesse cenário, a auriculoacupuntura como uma intervenção efetiva para o cuidado à saúde de indivíduos com alterações emocionais^(8)^. Esta é uma das 29 Práticas Integrativas e Complementares em Saúde (PICS) institucionalizadas no Sistema Único de Saúde (SUS) do Brasil, que são vistas como uma importante estratégia de assistência à saúde, especialmente ao considerarem a integralidade do ser humano e o seu caráter complementar aos tratamentos convencionais, além de proporcionarem acolhimento e escuta qualificada^(9)^. Diversos profissionais estão habilitados para a utilização das PICS, entre eles, os enfermeiros, conforme resolução do Conselho Federal de Enfermagem n^o^ 739/2024^(10)^, que recomenda a capacitação para a auriculoacupuntura por meio de cursos livres, com carga horária mínima de 80 horas.

Esta intervenção possui origem nos preceitos da Medicina Tradicional Chinesa, e é baseada na estimulação de pontos específicos no pavilhão auricular, a qual desencadeia modulações no Sistema Nervoso Central, para o reestabelecimento da homeostase^(11)^. Além disso, apresenta as vantagens de ter efeitos colaterais mínimos e ser de fácil operacionalização^(7,12)^.

As agulhas são os dispositivos mais comuns para aplicação da auriculoacupuntura^(13,14)^, sendo consideradas como o padrão ouro para aplicação da intervenção. Com a evolução tecnológica e com o objetivo de tornar a aplicação da técnica mais segura tanto para os clientes quanto para os profissionais, novos dispositivos de aplicação estão ganhando destaque, como o laser de baixa intensidade^(14)^. Quando empregado com baixa intensidade luminosa, a energia mínima da luz promove a ativação das zonas biologicamente reativas na orelha (os acupontos auriculares) e, consequentemente, o restabelecimento da homeostasia do organismo^(15)^. Dessa forma, a diminuição da invasividade, em comparação com a intervenção com o uso de agulhas, constitui um fator significativo que favorece a aceitação do tratamento empregando o laser^(16)^.

Nesse contexto, a fim de minimizar os riscos e promover um cuidado terapêutico tão eficaz quanto o da auriculoacupuntura com agulhas, além de abordar a potencialidade da laserterapia e a escassez de estudos utilizando o laser, sobretudo para o tratamento de desordens emocionais em estudantes universitários, este estudo propôs comparar a efetividade da auriculoacupuntura com laser e agulhas no tratamento da ansiedade em universitários no contexto pós pandêmico da Covid-19, bem como avaliar os possíveis sintomas ou reações adversas desencadeados pelas intervenções.

## MÉTODO

### Desenho do Estudo

Ensaio clínico randomizado, multicêntrico, paralelo, aberto, composto por dois grupos: auriculoacupuntura com agulhas (grupo agulha - GA - controle) e auriculoacupuntura com laser (grupo laser - GL - experimental), reportado segundo recomendações do Consolidated Standards of Reporting Trials (CONSORT)^(17)^.

### Local

O estudo foi realizado em duas universidades públicas do estado de Minas Gerais, entre o período de dezembro de 2023 e abril de 2024.

### População e Critérios de Seleção

A população do estudo foi composta por 1.093 estudantes universitários provenientes de todos os cursos de graduação das referidas universidades, das quatro áreas do conhecimento: ciências agrárias; ciências biológicas e da saúde; ciências exatas e tecnológicas; ciências humanas, letras e artes, sendo 62 cursos de uma universidade, distribuídos em três *campi*, e 40 da outra, também em três *campi*, com níveis moderados ou altos de ansiedade (ou seja, pontuação maior do que nove na *Depression, Anxiety and Stress Scale* (DASS-21)^(18)^. O estudo foi divulgado nos canais de comunicação oficial das universidades, nos *e-mails* institucionais e por meio de entrega de panfletos. Foram incluídos no estudo os estudantes com 18 anos ou mais e com disponibilidade de tempo para realização das avaliações e sessões de tratamento. Foram excluídos aqueles que não aceitaram ser alocados aleatoriamente nos dois grupos de estudo; apresentaram rejeição ou medo de receber as intervenções, histórico de alergia a fita microporosa ou ao metal, histórico de fotossensibilidade e de câncer de pele na região da cabeça e do pescoço, sistema imunológico comprometido, epilepsia, uso de *peeling* químico ou isotretinoína até seis meses anteriores ao início do tratamento, perfurações na orelha próximas aos pontos auriculares selecionados, uso de aparelhos auditivos ou marcapasso; tatuagens, lesões, inflamação ou deformidade no pavilhão auricular; gravidez; lactação; mulheres que planejaram engravidar durante o período do estudo; e aqueles que receberam alguma PICS nos últimos três meses.

Foram considerados, ainda, critérios de descontinuidade: a falta em alguma das avaliações; a perda de duas sessões de auriculoacupuntura consecutivas ou a ausência de mais de 10 dias entre as sessões; a apresentação de reação inflamatória na orelha; desconforto intenso no local de aplicação das agulhas ou do laser; ou manifestação do desejo de interromper a participação na pesquisa.

### Cálculo do Tamanho da Amostra e Randomização

O cálculo da amostra foi baseado na diferença de médias entre os grupos entre as avaliações inicial ( $\bar{\text{X}}$ = 2,71; σ^2^ = 9,22) e final ( $\bar{\text{X}}$ = 1,47; σ^2^ = 11,29), com relação ao nível de ansiedade, que foi avaliado por meio do Inventário de Ansiedade Estado (Idate-E)^(19)^. Foi utilizado o *software* RMASS2®: *Repeated Measures with Attrition:Sample Sizes for 2 Groups*, após realização de um teste piloto com 20 indivíduos (10 por grupo). Para um nível de significância de 5%, poder de 90% e tamanho de efeito médio (0.5), foi estimada uma amostra mínima de 40 indivíduos em cada grupo. A fim de garantir o tamanho amostral, o cálculo inicial foi corrigido em 30%, o que aumentou a amostra para 52 indivíduos por grupo.

A randomização dos grupos foi realizada por um pesquisador externo, na proporção 1:1, em bloco de 10 estudantes. Para a construção dos blocos, foi gerada uma lista de números aleatórios por meio do *site* (http://www.randomization.com/). Após a geração da lista, a sequência dos grupos foi cortada e colocada em envelope opaco, numerado e lacrado. Imediatamente antes da realização da primeira sessão de AA, o envelope com a randomização foi aberto pelo intervencionista e os estudantes foram alocados, de forma aleatória, para o grupo controle (auriculoacupuntura com agulhas – GA; n=61) ou para o grupo experimental (auriculoacupuntura com laser – GL; n=65).

Os participantes do estudo e os avaliadores não foram mascarados, devido às peculiaridades das intervenções aplicadas; ou seja, ambos poderiam identificar a alocação dos grupos pela presença ou não das agulhas semipermanentes no pavilhão auricular no GA.

### Intervenções e Coleta de Dados

As intervenções foram aplicadas por pesquisadores enfermeiros, com experiência de dois a 12 anos na aplicação da auriculoacupuntura.

As sessões foram realizadas com o intervalo de uma semana, alterando-se o pavilhão auricular a cada sessão e totalizando cinco sessões. Foram utilizados o mapa auricular preconizado pela *World Federation of Acupuncture-Moxibustion Societies* ^(20)^ e um localizador elétrico de acupontos (EL30 Finder NKL Basic® com caneta diferencial vermelha) para a precisa localização dos pontos auriculares. Foram aplicados nove pontos auriculares, previamente validados^(21)^: *Shenmen* (TF_4_), Sistema Nervoso Simpático (AH_6a_), Rim (CO_10_), Fígado (CO_12_), Baço (CO_13_), Tronco cerebral (AT_3,4i_), Pulmão 1 (CO_14_), Coração (CO_15_) e *Yang* do Fígado 2 (HX_8_ – porção inferior).

Foi realizada antissepsia do pavilhão auricular com algodão e álcool etílico a 70%. No GA, a auriculoacupuntura foi realizada com agulhas semipermanentes, esterilizadas e descartáveis, tamanho 0,20 × 1,5 milímetros (Complementar Agulhas®). No GL, foi utilizado laser de baixa intensidade de diodo, com comprimento de onda de 808 nanômetros (infravermelho), potência média de 100mW e área do ponto do feixe de 1 cm2 (Therapy ACP - DCM®). O laser foi aplicado em contato com a pele, de forma contínua, pontual (perpendicular à pele) e estacionária. O tempo de aplicação foi de 40 segundos para cada ponto estimulado, com a dose energética de 4 joules^(22)^ (irradiância por ponto: 100 mW/cm2; fluência por ponto: 4,0 J/cm2). Um espaçador (área: 0,09842 cm2; diâmetro: 3,54 mm - DMC®) foi utilizado na ponta da cânula do laser, com o objetivo de manter uma distância de segurança entre a cânula e a pele do participante. Antes da aplicação do laser, foi colocado filme de policloreto de vinila no espaçador e na cânula do equipamento para maior higiene, sendo trocado a cada sessão. Ainda, voluntário e terapeuta utilizaram óculos de proteção específicos para evitar possíveis danos à retina provocados pela luz laser.

Os participantes foram avaliados em três momentos: 1- Avaliação inicial (antes da primeira sessão de AA: os estudantes responderam, de forma *online*, o Instrumento de Caracterização Sociodemográfica e Psicossocial e o Idate-E^(19)^, e foram aferidos, por examinadores treinados, os parâmetros de frequências cardíaca e respiratória. 2- Avaliação final (uma semana após a quinta sessão): os participantes responderam, de forma *online*, ao Idate-E^(19)^ e ao questionário de satisfação e reações adversas, e foram aferidas as frequências cardíaca e respiratória pelos mesmos examinadores treinados. 3- Avaliação *follow-up* (em um período de seguimento de sete dias): os participantes responderam, *online*, ao Idate-E^(19)^ e novamente reavaliaram-se as frequências cardíaca e respiratória, pelos mesmos examinadores.

Para a caracterização sociodemográfica, investigou-se: idade; sexo; cor da pele autodeclarada; situação conjugal; moradia; atividade laboral; e renda familiar. O perfil clínico e psicossocial foi estabelecido por meio das seguintes variáveis: diagnóstico positivo de Covid-19; acompanhamento com psiquiatra e/ou psicólogo; diagnóstico clínico relacionado à saúde mental; momento de estabelecimento do diagnóstico; uso de psicofármacos e momento de início de uso; e autoavaliação das saúdes física e mental (em escala de zero a 10). Além disso, para investigar o perfil acadêmico, avaliaram-se área do curso, assistência estudantil, recebimento de bolsa e influência da pandemia da Covid-19 no desempenho acadêmico, na perspectiva de futuro profissional e na sobrecarga de atividades acadêmicas.

Foram investigados, ainda, os níveis de ansiedade, pelo Idate-E^(19)^ (desfecho primário), e os parâmetros de frequências cardíaca e respiratória (desfechos secundários).

O Idate-E é um questionário de autorrelato, em que se avalia o estado de ansiedade (estado emocional momentâneo)^(19)^. É constituído por vinte afirmativas, com quatro opções de respostas (1 -absolutamente não a 4 - muitíssimo)^(19)^. O escore varia de 20 a 80 pontos, sendo baixo grau de ansiedade (20–30), grau mediano de ansiedade (31–49) e grau elevado de ansiedade (≥ 50)^(23)^. Para esta avaliação, foi gerada uma versão *online* do questionário, via plataforma *Google Forms®,* que foi respondida em forma de autorrelato.

Para a avaliação das frequências cardíaca e respiratória, o participante permaneceu em repouso por 20 minutos, sentado em uma cadeira, com as costas apoiadas. A frequência cardíaca foi avaliada por um oxímetro digital portátil (Oxiled/LED G-TECH®) posicionado no dedo indicador da mão esquerda do participante, por um minuto, enquanto a frequência respiratória do participante foi mensurada por meio da observação de seu tórax ou abdômen, considerando a quantidade de vezes que realizou os movimentos combinados de inspiração e expiração em um minuto, sem que percebesse a intenção do avaliador, que utilizou um cronômetro.

Para avaliação do nível de satisfação com o tratamento, aplicou-se escala do tipo *likert*, de um a cinco pontos (1- extremamente insatisfeito a 5 -extremamente satisfeito); as possíveis reações adversas decorrentes das intervenções foram graduadas de zero (nenhum desconforto) a 10 (desconforto insuportável).

### Tratamento e Análise dos Dados

Os dados coletados foram agrupados em um banco de dados, utilizando-se o aplicativo Microsoft Office Excel (2021) e analisados com o programa estatístico *Statistical Package for the Social Sciences* (SPSS), versão 26.0, por um pesquisador mascarado em relação à alocação dos grupos.

A homogeneidade entre os dois grupos foi verificada por meio dos testes de MannWhitney e Qui-Quadrado, a 5% de significância, em relação às variáveis sociodemográficas, clínicas, psicossociais e acadêmicas.

Modelos de Equações de Estimativa Generalizada*/Generalized Estimating Equation* (GEE) foram utilizados para estimar e testar o efeito da alocação dos grupos, ajustando para o efeito do tempo (avaliações inicial, final e *follow up*) e interação (grupo *tempo), nas variáveis de desfecho. As variáveis foram tratadas assumindo distribuição gama e função de ligação logarítmica. O modelo com o menor *Quasi Likelihood Under Independence Model Criterion* (QIC) e o melhor ajuste foi considerado como o modelo final (não estruturado). A significância estatística foi estabelecida em p < 0,05. O teste post-hoc de Bonferroni foi usado para comparar as médias das correlações significativas entre os preditores e os desfechos.

### Aspectos Éticos

O estudo foi aprovado por Comitê de Ética em Pesquisa sob os pareceres número 5.700.107/ CAAE: 63318222. 0.0000.5153. Todos os estudantes que concordaram em participar do estudo assinaram o Termo de Consentimento Livre e Esclarecido em formato digital. Ao final do estudo, todos os participantes foram convidados a realizarem as mesmas sessões de tratamento do grupo em que não foram alocados inicialmente.

## RESULTADOS

Inicialmente, 197 estudantes foram avaliados para elegibilidade e 71 não atenderam aos critérios de inclusão. A amostra elegível obtida foi de 126 estudantes, os quais foram considerados para a randomização (61 no GA e 65 no GL) e 106 concluíram o estudo. Houve uma perda de 20 estudantes (15,85%) ao longo do estudo, os quais foram excluídos da análise estatística, uma vez que o tamanho amostral foi atingido (Figura 1).

**Figura 1:** Fluxograma de rastreamento da amostra.

Fonte: Adaptado segundo o modelo Consort(17).

Nota: *n = frequência absoluta; ^†^GA = Grupo Agulha (controle); ‡GL = Grupo Laser (intervenção).

Os dados da Tabela 1 apresentam a comparação entre os grupos conforme o perfil sociodemográfico, clínico, psicossocial e acadêmico. Não houve diferenças estatisticamente significativas entre os grupos em relação a estas variáveis, o que aponta para a homogeneidade entre eles.

**Tabela 1:** Caracterização da amostra quanto ao perfil sociodemográfico, clínico, psicossocial e acadêmico – Viçosa, MG, Brasil, 2024. (n = 106).

Perfil sociodemográfico				
Variáveis		*GA (n = 53)	^†^GL (n = 53)	Valor p
Idade (^‡^ $\bar{\text{X}}$ ^§^dp)	Anos	22,83 ± 4,13	22,04 ± 4,98	0,700**
Sexo	Masculino	16 (30,2)	10 (18,9)	0,176 ^††^
^||^n (^¶^%)	Feminino	37 (69,8)	43 (81,1)	
Cor da pele autodeclarada	Branco	30 (56,6)	32 (60,4)	0,764^††^
^||^n (^¶^%)	Amarelo	0 (0,0)	0 (0,0)	
	Pardo	16 (30,2)	16 (30,2)	
	Preto	6 (11,3)	5 (9,4)	
	Indígena	1 (1,9)	0 (0,0)	
Situação conjugal	Solteiro	45 (84,9)	44 (83,0)	0,603^††^
^||^n (^¶^%)	Casado/ União estável	8 (15,1)	8 (15,1)	
	Viúvo	0 (0,0)	1 (1,9)	
	Divorciado	0 (0,0)	0 (0,0)	
Moradia	Sozinho	7 (13,2)	9 (17,0)	0,599^††^
^||^n (^¶^%)	Com família	18 (34,0)	13 (24,5)	
	República	24 (45,3)	24 (45,3)	
	Alojamento estudantil	4 (7,5)	7 (13,2)	
Atividade laboral	Não	47 (88,7)	46 (86,8)	0,767^††^
^||^n (^¶^%)	Sim	6 (11,3)	7 (13,2)	
Renda familiar	1 a 1 e ½ salários mínimos	16 (30,2)	15 (28,3)	0,465^††^
^||^n (^¶^%)	2 a 3 salários mínimos	22 (41,5)	19 (35,8)	
	4 a 5 salários mínimos	11 (20,8)	12 (22,6)	
	6 ou mais salários mínimos	4 (7,5)	7 (13,2)	
**Perfil clínico e psicossocial**				
Diagnóstico positivo para Covid-19	Não	30 (56,6)	30 (56,6)	1,000^††^
^||^n (^¶^%)	Sim	23 (43,4)	23 (43,4)	
Acompanhamento com psiquiatra	Não	34 (64,2)	33 (62,3)	0,884^††^
^||^n (^¶^%)	Já fazia antes da pandemia	7 (13,2)	6 (11,3)	
	Começou a fazer durante/após a pandemia	12 (22,6)	14 (26,4)	0,495^††^
Acompanhamento com psicólogo	Não	27 (50,9)	26 (49,1)	0,455^††^
^||^n (^¶^%)	Já fazia antes da pandemia	12 (22,6)	8 (15,1)	
	Começou a fazer durante/após a pandemia	14 (26,4)	19 (35,8)	0,150^††^
Diagnósticos clínicos relacionados à saúde mental	Depressão	10 (18,9)	9 (17,0)	0,800^††^
^||^n (^¶^%)	Ansiedade	27 (50,9)	21 (39,6)	0,242^††^
	Síndrome do Estresse Pós traumático	0 (0,0)	1 (1,9)	0,315^††^
	Síndrome do pânico	2 (3,8)	0 (0,0)	0,153^††^
	Transtornos do humor	1 (1,9)	2 (3,8)	0,558^††^
	Transtorno obsessivo compulsivo	0 (0,0)	2 (3,8)	0,153^††^
	Transtorno do déficit de atenção e hiperatividade	1 (1,9)	1 (1,9)	1,000^††^
	Transtornos de personalidade	0 (0,0)	1 (1,9)	0,315^††^
	Fobia social	1 (1,9)	1 (1,9)	1,000^††^
	Transtornos/distúrbios do sono	3 (5,7)	3 (5,7)	1,000^††^
Momento do diagnóstico clínico relacionado à saúde mental	Não possui nenhum diagnóstico	18 (34,0)	22 (41,5)	0,638^††^
^||^n (^¶^%)	Já possuía o diagnóstico anteriormente à pandemia	18 (34,0)	14 (26,4)	
	Estabelecido durante a pandemia	17 (32,1)	17 (32,1)	
Uso de psicofármacos	Antidepressivo	19 (35,8)	18 (34,0)	0,839^††^
^||^n (^¶^%)	Ansiolítico	19 (35,8)	17 (32,1)	0,682^††^
	Antipsicótico	1 (1,9)	3 (5,7)	0,308^††^
	Estabilizador do humor	1 (1,9)	3 (5,7)	0,308^††^
	Hipnótico não benzodiazepínico	1 (1,9)	0 (0,0)	0,315^††^
	Anfetaminas	1 (1,9)	0 (0,0)	0,315^††^
Momento de início de uso do psicofármaco	Não faz uso	30 (56,6)	30 (56,6)	0,957^††^
^||^n (^¶^%)	Antes da pandemia	11 (20,8)	10 (18,9)	
	Depois da pandemia	12 (22,6)	13 (24,5)	
Autoavaliação da saúde física (^‡^ $\bar{\text{X}}$ ^§^dp)	zero a 10	5,34 ± 2,12	5,71 ± 2,04	0,467**
Autoavaliação da saúde mental (^‡^ $\bar{\text{X}}$ ^§^dp)	zero a 10	4,33 ± 1,63	4,45 ± 1,74	0,819**
**Perfil acadêmico**				
Área do curso	Ciências Agrárias	9 (17,0)	10 (18,9)	0,982^††^
^||^n (^¶^%)	Ciências Biológicas e da Saúde	23 (43,4)	22 (41,5)	
	Ciências Exatas e Tecnológicas	9 (17,0)	10 (18,9)	
	Ciências Humanas, Artes e Letras	12 (22,6)	11 (20,8)	
Recebe assistência estudantil	Não	38 (71,7)	32 (60,4)	0,218^††^
^||^n (^¶^%)	Sim	15 (28,3)	21 (39,6)	
Recebe bolsa de ensino, pesquisa ou extensão	Não	40 (75,5)	41 (77,4)	0,819^††^
^||^n (^¶^%)	Sim	13 (24,5)	12 (22,6)	
Desempenho acadêmico	Não mudou	9 (17,0)	4 (7,5)	0,171^††^
^||^n (^¶^%)	Melhorou	3 (5,7)	7 (13,2)	
	Piorou	41 (77,4)	42 (79,2)	
Perspectiva de futuro profissional	Não mudou	16 (30,2)	13 (24,5)	0,258^††^
^||^n (^¶^%)	Melhorou	5 (9,4)	11 (20,8)	
	Piorou	32 (60,4)	29 (54,7)	
Sobrecarga de atividades acadêmicas após a pandemia da Covid-19	Não	5 (9,4)	5 (9,4)	1,000^††^
^||^n (^¶^%)	Sim	48 (90,6)	48 (90,6)	

Nota:

*GA = Grupo Agulha (controle)

^†^GL = Grupo Laser (experimental)

^‡^ $\bar{\text{X}}$ = média amostral

^§^dp = desvio padrão

^||^n = frequência absoluta

^¶^% = frequência relativa

** = Mann-Whitney

^††^ = Qui-quadrado de Pearson.

A Tabela 2 apresenta os níveis de ansiedade na amostra estudada. Inicialmente, os grupos eram homogêneos em relação a essa variável, uma vez que não houve diferença estatisticamente significativa entre eles. Observou-se redução estatisticamente significativa nos GA e GL, entre as avaliações inicial e final (diferença de média: 9,34 e 7,83 respectivamente), e inicial e *follow up* (diferença de média: 7,81 e 6,53, respectivamente). O nível de ansiedade permaneceu constante entre as avaliações final e *follow up* em ambos os grupos, ou seja, a redução alcançada no final do tratamento permaneceu por sete dias após o término dele. Não houve diferença estatisticamente significativa entre os grupos nas avalições final e *follow up*, o que confirma que a aplicação da auriculoacupuntura com agulhas ou com laser possui o mesmo efeito.

**Tabela 2:** Análise do nível de ansiedade, expressa em média, erro padrão e intervalo de confiança à 95% – Viçosa, MG, Brasil, 2024. (n = 106).

Ansiedade	Inicial	Final	Follow up	Valor p*IC 95%		
	^†^ $\bar{\text{X}}$ (erro padrão)	^†^ $\bar{\text{X}}$ (erro padrão)	^†^ $\bar{\text{X}}$ (erro padrão)	Inicial–Final	Final–Follow up	Inicial–Follow up
^‡^GA (^§^n = 53)	58,85 (1,215)	49,51 (1,436)	51,04 (1,942)	**<0,0015,93; 12,74**	0,801 -4,83; 1,77	**<0,0013,76; 11,86**
^||^GL (^§^n = 53)	57,87 (1,193)	50,04 (1,408)	51,34 (1,435)	**<0,0014,56; 11,10**	0,921 -4,35; 1,75	**<0,0012,87; 10,19**
Valor p	1,000	1,000	1,000			
*IC 95%	-4,02;5,98**	-6,43;5,38**	-7,39;6,79**			

Nota:

*IC 95% = Intervalo de confiança para a diferença de médias em 95%

^†^ $\bar{\text{X}}$ = média amostral

^‡^GA = Grupo Agulha (controle)

^§^n = frequência absoluta

^||^GL = Grupo Laser (experimental)

** = Diferença de médias: GA – GL.

A Tabela 3 apresenta os dados das frequências cardíaca e respiratória dos participantes do estudo. Inicialmente, os grupos eram homogêneos em relação a essas duas variáveis. Em relação à frequência cardíaca, observou-se redução estatisticamente significativa no GA entre as avaliações final e *follow up* (diferença de média: 3,94) e entre as avaliações inicial e *follow up* (diferença de média: 4,38). Já o GL permaneceu constante ao longo do tempo. Ainda, os grupos não diferenciaram entre si nas avaliações final e follow up, demostrando os mesmos efeitos exercidos pelas aplicações da agulha e do laser.

**Tabela 3:** Análise das frequências cardíaca e respiratória, expressas em média, erro padrão e intervalo de confiança à 95% – Viçosa, MG, Brasil, 2024. (n = 106).

	Frequências	Inicial	Final	Follow up	Valor p*IC 95%		
		^†^ $\bar{\text{X}}$ (erro padrão)	^†^ $\bar{\text{X}}$ (erro padrão)	^†^ $\bar{\text{X}}$ (erro padrão)	Inicial–Final	Final–Follow up	Inicial–Follow up
**Cardíaca**	^‡^GA (^§^n = 53)	83,91 (1,954)	83,47 (1,517)	79,53 (1,496)	1,000 -3,80; 4,67	**0,022 0,33; 8,42**	**0,029 0,42; 7,47**
	^||^GL (^§^n = 53)	81,62 (1,552)	83,09 (1,263)	83,40 (1,418)	1,000 -5,61; 2,67	1,000 -3,62; 3,02	0,912 -5,90; 2,36
	Valor p	1,000	1,000	0,908			
	*IC 95%	-5,04; 9,91**	-5,42; 6,17**	-9,92; 2,18**			
**Respiratória**	^‡^GA (^§^n = 53)	16,77 (0,440)	16,47 (0,409)	15,60 (0,440)	1,000 -0,84; 1,45	0,168 -0,22; 1,96	0,054 -0,01; 2,35
	^||^GL (^§^n = 53)	15,79 (0,358)	15,68 (0,312)	15,68 (0,418)	1,000 -0,78; 1,01	1,000 -0,89; 0,89	1,000 -1,01; 1,23
	Valor p	1,000	1,000	1,000			
	*IC 95%	-0,68; 2,65**	-0,72; 2,30**	-1,86; 1,71**			

Nota:

*IC 95% = Intervalo de confiança para a diferença de médias em 95%

^‡^ $\bar{\text{X}}$ = média amostral

^‡^GA = Grupo Agulha (controle)

^§^n = frequência absoluta

^||^GL = Grupo Laser (experimental)

** = diferença de médias: GA - GL.

Referentemente à frequência respiratória, não houve alterações estatisticamente significativas ao longo do tempo, em ambos os grupos. Ademais, os grupos não diferenciaram estatisticamente entre si nas avaliações final e *follow up,* confirmando a igualdade de efeito entre as intervenções (Tabela 3).

No que tange ao nível de satisfação dos participantes em relação ao tratamento realizado, destaca-se que 39,6% (n = 21) dos participantes no GA e 41,5% (n = 22) no GL ficaram extremamente satisfeitos; 41,5% (n = 22) no GA e 37,7% (n = 20) no GL ficaram satisfeitos; 15,1% (n = 8) no GA e 17,0% (n = 9) no GL não tinham certeza; nenhum participante no GA e 1,9% (n = 1) no GL ficaram insatisfeitos; e 3,8% (n = 2) e 1,9% (n = 1) ficaram extremamente insatisfeitos, respectivamente, com valor p = 0,825 segundo teste de Qui-quadrado de Pearson.

A Tabela 4 apresenta a frequência e a intensidade dos sintomas ou reações adversas. A dor devido à permanência das agulhas na orelha, a hiperemia, o prurido, o edema e a descamação foram mais frequentes no GA. Em relação à intensidade dos sintomas, apenas cefaleia e rubor na orelha não diferenciaram entre os grupos; os demais foram mais intensos no GA em relação ao GL, mas classificados como leves.

**Tabela 4:** Frequência e intensidade dos sintomas ou reações adversas dos participantes do estudo que receberam auriculoacupuntura com agulhas e com laser, Viçosa, MG, Brasil, 2024. (n = 106).

Sintoma ou reação adversa	Frequência *n (^†^%)		Valor p
	^‡^GA (*n = 53)	^§^GL (*n = 53)	
Dor na orelha	46 (86,8)	06 (11,3)	<0,001**
Hiperemia na orelha	31 (58,5)	10 (18,9)	<0,001**
Prurido na orelha	29 (54,7)	08 (15,1)	<0,001**
Cefaleia relacionada à intervenção	19 (35,8)	17 (32,1)	0,682**
Edema na orelha	16 (30,2)	00 (0,00)	<0,001^††^
Descamação na orelha	12 (22,6)	03 (5,70)	0,023^††^
Linfonodos aumentados	08 (15,1)	02 (3,80)	0,093^††^
Rubor na orelha	00 (0,00)	01 (1,90)	1,000^††^
**Intensidade (escala de zero a 10) ^|^ $\bar{\text{X}}$ (^^¶^^dp**)			
Dor na orelha	3,23 (2,44)	0,19 (0,65)	<0,001^††^
Hiperemia na orelha	1,89 (2,18)	0,23 (0,50)	<0,001^††^
Prurido na orelha	2,28 (2,77)	0,28 (0,74)	<0,001^††^
Cefaleia relacionada à intervenção	1,30 (2,39)	1,11 (1,91)	0,761^††^
Edema na orelha	0,74 (1,51)	0,00 (0,00)	<0,001^††^
Descamação na orelha	0,66 (1,55)	0,11 (0,50)	0,012^††^
Linfonodos aumentados	0,55 (1,46)	0,13 (0,73)	0,048^††^
Rubor na orelha	0,00 (0,00)	0,02 (0,14)	0,317^††^

Nota:

*n = frequência absoluta

^†^% = frequência relativa

^‡^GA = Grupo Agulha (controle)

^§^GL = Grupo Laser (experimental)

^||^ $\bar{\text{X}}$ = média amostral

^¶^dp = desvio padrão

** = Qui-quadrado de Pearson

^††^ = Exato de Fisher

^‡‡^ = Mann-Whitney.

## DISCUSSÃO

Os resultados deste estudo demostraram que a auriculoacupuntura realizada com laser possui efetividade semelhante à auriculoacupuntura realizada com agulhas semipermanentes para o tratamento de ansiedade em universitários no contexto pós pandêmico da Covid-19. Além disso, evidenciou-se maior ocorrência de sintomas ou reações adversas no grupo que recebeu a intervenção com agulhas, apesar da intensidade leve.

A utilização das agulhas na auriculoacupuntura já é consolidada no meio científico. De fato, evidências científicas^(21,24)^ já apontam a efetividade da intervenção com agulhas no tratamento de desordens emocionais. Esses são os dispositivos da acupuntura mais tradicionais e com efetividade comprovada. Já no que se refere à utilização do laser em desordens emocionais, as evidências científicas ainda são incipientes e há diversidade de parâmetros dosimétricos adotados, o que representa uma área que necessita de mais investigações.

Destaca-se, até o presente momento, o uso do laser na ansiedade^(25)^, no sono^(25)^, em desordens temporomandibulares^(25)^ e na depressão associada a esta condição^(22)^. Fernandes e colaboradores^(25)^ realizaram 10 sessões semanais, com o laser infravermelho de baixa intensidade, comprimento de onda de 808 nm (infravermelho), com potência de 100 mW, nos pontos auriculares *Shenmen,* Membro Superior, Zero, Estômago, Maxila, Mandíbula, Ansiedade e Estresse, e encontraram que a auriculoterapia foi eficaz no tratamento da ansiedade; no entanto, não se mostrou eficaz nos resultados do distúrbio do sono e sintomas de disfunção temporomandibular. Já Rodrigues e colaboradores^(22)^ realizaram a aplicação do laser nos pontos *Shenmen,* Articulação temporomandibular e Coração, uma vez por semana, durante oito sessões. O laser utilizado foi o de diodo pulsado de 75 W (InGaAs\GaAs), potência de saída de 50 mW, comprimento de onda de 904 nm (infravermelho), a 4 J/cm2 durante por 24 segundos em cada ponto auricular irradiado^(22)^. Os autores encontraram que a auriculoterapia a laser de baixa potência melhorou os sintomas físicos e emocionais da disfunção temporomandibular, com resultados semelhantes ao grupo que utilizou placas oclusais^(22)^. Diante do exposto, evidencia-se que esta é uma área que ainda necessita investigação, uma vez que há poucas evidências científicas na temática, não há padronização em relação aos parâmetros dosimétricos e os resultados ainda são divergentes entre os estudos.

Nesse contexto de cuidados de saúde a indivíduos com alterações emocionais, o presente estudo contribui com mais uma evidência científica sobre a efetividade do laser, o que reforça a sua utilização como uma estratégia não farmacológica para o alívio da ansiedade. Assim, a implementação desta terapêutica no ambiente universitário poderá promover aos estudantes o acesso a essa PICS efetiva, realizada por meio de um recurso tecnológico seguro e de fácil execução, mas que ainda não está totalmente disponível nas redes de atenção à saúde do SUS. Isso poderá contribuir com a promoção da saúde mental dos estudantes e, consequentemente, favorecer o melhor desempenho acadêmico e uma perspectiva de futuro profissional mais positiva.

Destaca-se, ainda, que as PICS se constituem como importantes estratégias de cuidado, ao centralizar a atenção no usuário e nas suas demandas, proporcionando uma assistência ampliada, resolutiva e integral^(26)^.

No contexto da enfermagem^(10)^, o enfermeiro pode implementar a auriculoacupuntura, com agulha ou com laser, na sua prática clínica, como intervenção efetiva para o tratamento e a prevenção da ansiedade, que é um problema de enfermagem sob sua responsabilidade.

Além da redução da ansiedade, o presente estudo também avaliou outros parâmetros, como as frequências cardíaca e a respiratória. Apesar de uma redução da frequência cardíaca entre os participantes submetidos à auriculoacupuntura com agulhas, ao comparar ambas as intervenções, não houve diferença estatisticamente significativa entre elas ao final do tratamento.

Tais achados relacionados à frequência cardíaca estão em consonância com estudo que avaliou os efeitos da auriculoacupuntura com agulha no nervo vago sobre o sistema nervoso autônomo cardíaco^(27)^. No presente estudo, sugere-se que a estimulação realizada nos pontos localizados nas conchas cimba e cava pode ter desencadeado a estimulação do nervo vago, de modo a gerar alterações na frequência cardíaca^(28)^. Além disso, pelo fato de as agulhas promoverem dor e resposta microinflamatória local^(29)^, os pontos permanecem reativos, o que torna as mudanças mais perceptíveis no grupo agulha.

Em contrapartida, no presente estudo não ocorreram alterações estatisticamente significativas na frequência respiratória em ambos os grupos. Esse achado contrapõe-se ao de um ensaio clínico com o objetivo de avaliar o efeito da auriculoterapia na ansiedade e nos parâmetros fisiológicos de pacientes submetidos à angiografia coronária^(30)^. A intervenção foi aplicada por meio da acupressão leve, nos acupontos Shenmen, Relaxamento, Tranquilizante e Endócrino no lado não dominante, sendo cada um deles estimulado com o polegar por um minuto no sentido anti-horário, 60 minutos antes da angiografia. A frequência respiratória foi avalida por meio das observações dos movimentos do tórax pelo mesmo pesquisador, por um minuto^(30)^. Sugere-se que os efeitos sobre a frequência respiratória divergiram dos observados no presente estudo em virtude do uso de outros pontos auriculares terem sido utilizados, como Endócrino, Relaxamento e Tranquilizante^(30)^, que também possuem ação na ansiedade.

No que diz respeito aos sintomas ou reações adversas, a dor na orelha foi o sintoma mais comum e intenso relatado pelos participantes do grupo agulha. Porém, a gravidade foi considerada como sendo de baixa intensidade. Isso indica que, apesar de efeitos adversos, os estudantes costumam tolerar bem a auriculoacupuntura com agulhas^(7,12)^, visto que as reações não foram graves.

Por outro lado, a utilização do laser demonstrou ser vantajosa ao considerar que os resultados são equiparáveis ao uso das agulhas em relação aos níveis de ansiedade, que é o padrão ouro, bem como apresentou reações menos intensas. Destaca-se, ainda, que o laser é um dispositivo não invasivo, que não apresenta risco de infecção, é indolor, de fácil e rápida aplicação e, principalmente, benéfico para pessoas com fobia a agulhas^(14,15)^. É importante destacar que reações adversas muito raramente acontecem na auriculoacupuntura. Reações fatais ou eventos adversos irreversíveis não foram documentados na literatura cientifica até o momento. Eventos adversos menores, como dor local, sangramento ou infecções podem ocorrer após o uso de agulhas, que são preveníveis quando técnicas corretas de desinfecção dos materiais, antissepsia e inspeção constante do pavilhão auricular são adotadas^(14)^. Salienta-se, também, que, até onde se sabe, a literatura científica ainda não apresenta dados comparando o laser à agulha em relação ao potencial de infecção das intervenções, o que evidencia o caráter inovador do presente estudo. Quanto à satisfação dos participantes em relação às intervenções recebidas, ambos os grupos se mostraram satisfeitos. Isso confirma a segurança e a eficácia da técnica.

Como limitações desse estudo, destaca-se, primeiramente, a ausência de mascaramento dos participantes, que foi impossibilitado devido às peculiaridades das intervenções aplicadas. Apesar disso, os intervencionistas foram diferentes dos avaliadores para evitar qualquer interferência nos dados coletados, bem como o profissional que realizou as análises dos resultados desconhecia a alocação dos grupos. Em segundo lugar, destaca-se a perda amostral. Entretanto, esta não foi expressiva e o número de estudantes atingiu o valor estimado pelo cálculo amostral, o que confirma a magnitude dos achados. Outro fator que merece destaque é a avaliação do desfecho frequência respiratória mediante observação, uma vez que pode ter sofrido interferência do avaliador.

Apesar das limitações, a efetividade demonstrada neste estudo da auriculoacupuntura com agulhas e com laser contribui para fortalecer a crescente base de evidências que apoia a integração desta intervenção como uma opção terapêutica complementar no tratamento da ansiedade, a qual pode ser adotada pelo enfermeiro na sua prática clínica, de forma a ampliar o seu escopo de atuação. Ademais, incentiva-se o uso da auriculoacupuntura, tanto com agulhas como com laser, para o manejo da ansiedade em universitários pelas instituições de ensino superior, de maneira a repercutir, possivelmente, no desempenho acadêmico e na qualidade de vida, com possíveis impactos na redução do consumo de psicofármacos, que podem causar dependência, e do abuso de álcool e outras drogas. Recomenda-se, para pesquisas futuras, estudos com amostras maiores para elucidar os efeitos da auriculoacupuntura com ambos os dispositivos nos parâmetros vitais que podem sofrer alteração em virtude da presença da ansiedade, além de outros desfechos que podem estar associados à ansiedade, como regulação do humor prejudicada, tristeza crônica, risco de comportamento suicida, sobrecarga de estresse, dentre outros, que também são de responsabilidade do enfermeiro.

## CONCLUSÃO

Tanto a auriculoacupuntura com agulhas quanto com laser foram efetivas na redução dos níveis de ansiedade em universitários, com efeitos observados sete dias após o término do tratamento. Contudo, não houve diferença estatisticamente significativa entre os grupos em relação aos parâmetros de frequências cardíaca e respiratória, apesar da redução da frequência cardíaca ao longo do tempo no grupo que foi tratado com agulhas. Além disso, evidenciou-se que o uso do laser na auriculoacupuntura é vantajoso quando se consideram as reações adversas apresentadas pelas agulhas.

## Data Availability

https://doi.org/10.48331/scielodata.DX72SY
